# The Double Bell's: Unraveling Idiopathic Bilateral Facial Paralysis in a 31-Year-Old

**DOI:** 10.7759/cureus.69093

**Published:** 2024-09-10

**Authors:** Aditya Jain, Rajeshwar Ranganathan, Abhishek Sinha, Abizar Rangoonwala, Subramaniam Kohul

**Affiliations:** 1 Emergency Medicine, United Lincolnshire Hospitals NHS Trust, Boston, GBR

**Keywords:** bells palsy, bilateral bell's palsy, facial asymmetry, facial nerve paralysis, ramsay hunt syndrome

## Abstract

Bell's palsy, an acute, idiopathic, and typically unilateral facial nerve paralysis, represents a common cause of sudden facial weakness. The aetiology is often attributed to viral infections. This case report discusses the presentation, diagnosis, and management of a rare case of idiopathic bilateral Bell's palsy. We present a case of a 31-year-old male who presented to the emergency department with a one-week history of progressive bilateral facial weakness following initial neck and jaw pain. Despite the resolution of pain, the patient experienced complete facial paralysis on both sides, including the inability to raise eyebrows, close eyes fully, and numbness over the lips. The patient presented with no complaints of headache, trauma, vision changes, or recent travel history. Examination and routine blood tests yielded normal results, and a head CT scan showed no abnormalities. As a result, the diagnosis of idiopathic bilateral Bell's palsy was confidently confirmed. This case highlights the clinical presentation, diagnostic approach, and management of a rare bilateral facial palsy, emphasizing the importance of a comprehensive evaluation and considering Bell's palsy in differential diagnoses of acute facial weakness.

## Introduction

Bell's palsy is an acute condition marked by a sudden, unilateral facial nerve paralysis of unknown aetiology. Diagnosis is primarily based on exclusion and thorough physical examination. Bilateral facial nerve palsy incidence is exceptionally low, accounting for less than 2% of all facial palsy cases [1]. Even though Bell's palsy is typically characterized by one-sided facial nerve involvement, it is uncommon for both sides to be affected. Bilateral facial nerve palsy is an exceedingly rare condition that involves the simultaneous or sequential paralysis of both facial nerves within a short timeframe, usually around 30 days [1].

This case report presents a striking example of idiopathic bilateral Bell's palsy in a 31-year-old male. It elucidates this rare condition's clinical presentation, diagnostic process, and therapeutic management. The rarity of bilateral Bell's palsy necessitates a comprehensive diagnostic approach to exclude other potential aetiologies, highlighting the importance of recognizing and treating this unusual presentation promptly to prevent long-term complications. Through detailed examination and strategic management, this case underscores the critical need for vigilance in atypical presentations of facial nerve paralysis, emphasizing a multidisciplinary approach to ensure optimal patient outcomes.

## Case presentation

A 31-year-old male presented to the emergency department with a one-week history of gradually progressive bilateral facial weakness. The initial symptoms included pain on the left side of the neck and jaw, for which the patient was empirically commenced on antibiotics. A dentist evaluated the patient and ruled out any dental infection. Despite the resolution of pain, the patient subsequently developed complete facial paralysis on both sides, manifesting as an inability to raise his eyebrows, close his eyes fully, and numbness in his lips. He reported no headaches, trauma, visual changes, or recent travel history. He could swallow normally but had difficulty chewing.

**Figure 1:**
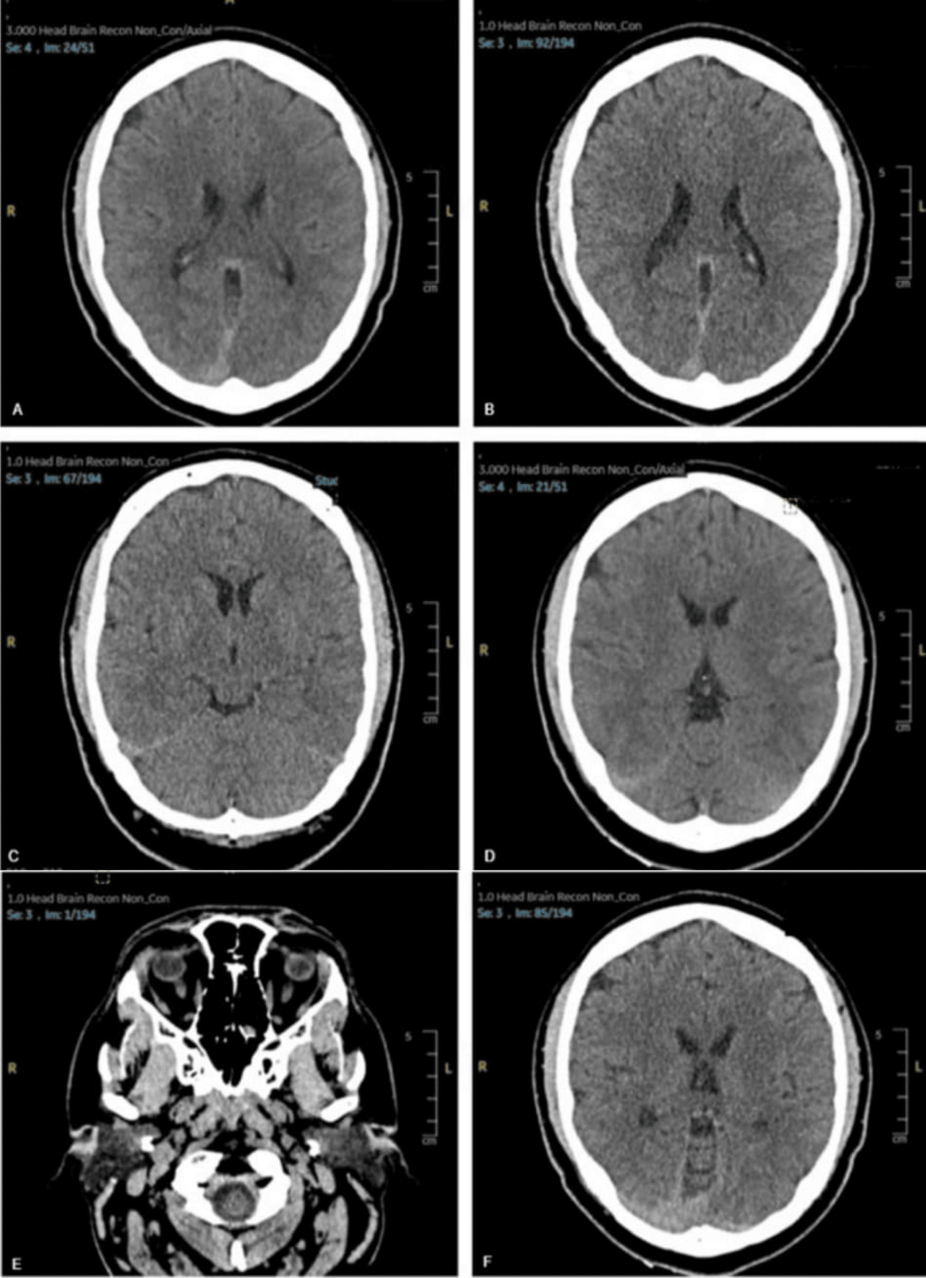
CT Imaging A-F: Images showing various sections of the patient's CT brain.

The patient was vitally stable on examination, with no abnormalities other than facial weakness. Neurological examination revealed complete bilateral facial paralysis without any other focal deficits. Routine blood tests were within normal limits, including a complete blood count, electrolytes, and inflammatory markers (Table 1). The patient's CT scan of the head showed no abnormalities (Figure 1).

**Table 1:** Investigations GFR: Glomerular filtration rate; CRP: C-reactive protein; HCV: Hepatitis C virus; ACE: Angiotensin-converting enzyme; ANA: Antinuclear antibody; c-ANCA: Cytoplasmic antineutrophil cytoplasmic antibody; p-ANCA: Perinuclear antineutrophil cytoplasmic antibody.

Investigation	Unit	Reference Ranges	Patient Values
Sodium	mmol/L	133-146	139
Potassium	mmol/L	3.5-5.3	4.4
Urea	mmol/L	2.5-7.8	4.7
Creatinine	umol/L	45-84	81
GFR	mL/min	90-200	>90
Serum Glucose	mmol/L	3-6	5.2
Haemoglobin	g/dL	117-149	166
White Blood Cells	10^9^/L	4.3-11.2	10.0
CRP	mg/dl	0-5	1.5
Lactate	mmol/L	0.5-2.2	5.8
Adjusted calcium	mmol/L	2.2-2.6	2.39
HIV			Negative
HBsAg			Negative
HCV			Negative
ACE Levels	IU/L	16-85	Normal
ANA			Negative
c-ANCA			Negative
p-ANCA			Negative
Anti-dsDNA			Negative

The severity of facial nerve weakness can be evaluated through the House-Brackmann Facial Nerve Grading System, which ranges from grade I (indicating no weakness) to grade VI (indicating total weakness). In cases of Bell's palsy, additional laboratory tests or imaging studies may not be necessary. However, if atypical symptoms are present, it is crucial to conduct further investigations to rule out a potential central cause of the symptoms [2-4].

Differential diagnosis

It can be caused by various factors, including infectious, neoplastic, degenerative, or neurological disorders [5]. Instances have been recorded where bilateral facial nerve paralysis was associated with illnesses such as syphilis, chickenpox, early HIV infection, and even advanced breast cancer [6-8]. These diverse aetiologies highlight the importance of a thorough diagnostic workup when encountering bilateral facial nerve palsy to identify the underlying cause and provide appropriate treatment.

Certain medical conditions, such as Heerfordt's syndrome, neurosarcoidosis, and Wegener's granulomatosis, have been linked to bilateral facial nerve paralysis in certain cases [9-11]. These associations emphasize the need for a multidisciplinary approach involving specialists from various fields, such as neurology, rheumatology, and oncology, to effectively manage cases of bilateral facial nerve palsy. Also, uncommon genetic conditions like Moebius syndrome may present with the participation of both facial nerves, underscoring the importance of genetic testing and counselling in particular instances [12].

Additionally, bilateral paralysis of the facial nerves may manifest in the presence of systemic conditions such as HIV/AIDS, lymphoma, and Burkitt lymphoma. This highlights the necessity of conducting a thorough assessment to manage both the neurological manifestations and the fundamental systemic ailment [13-15]. The possibility of complications like recurrent palsy or synkinesis underscores the significance of long-term follow-up and rehabilitation in these patients [16-17].

Given the patient's presentation and the normal findings on laboratory and imaging studies, a diagnosis of idiopathic bilateral Bell's palsy was established.

Management and outcome

The patient was treated with a course of oral acyclovir and prednisolone. Acyclovir was administered to address potential viral causes, particularly herpes simplex virus, which is implicated in Bell's palsy. Prednisolone was used to reduce inflammation and oedema of the facial nerve, aiming to expedite recovery. Diagnostic imaging modalities such as magnetic resonance imaging (MRI) play a crucial role in evaluating cases of bilateral facial nerve palsy, aiding in the identification of underlying pathologies and guiding treatment decisions [18-19]. Moreover, electrophysiological studies can help localize lesions and assess nerve function in patients with bilateral facial nerve palsy, contributing valuable information for prognosis and management [8].

The patient was educated about the nature of Bell's palsy and the expected course of the disease. He was advised to perform regular facial exercises to maintain muscle tone. Additionally, he was instructed on eye care measures, such as using artificial tears and eye patches to prevent corneal damage due to incomplete eye closure.

Follow-up appointments were arranged with ENT and ophthalmology specialists to monitor the progression of the palsy and manage any complications. At the follow-up visit, the patient showed partial improvement in facial movements, with better control over eyelid closure and mouth movements. He recovered gradually over the next few months, with significant improvement noted at six months post-diagnosis.

## Discussion

Bell's palsy is characterized by the sudden onset of unilateral facial weakness due to compression of the seventh cranial nerve at the geniculate ganglion [4]. Pathogenesis is believed to involve viral reactivation, leading to inflammation and subsequent facial nerve compression within its bony canal. The condition is generally self-limiting, with a significant proportion of patients recovering fully within six months.

In this case, the patient's presentation was consistent with idiopathic bilateral Bell's palsy, particularly given the preceding neck and jaw pain, which might suggest a viral aetiology. The lack of systemic symptoms and normal diagnostic tests further supported this diagnosis. The treatment with acyclovir aimed to address potential viral causes, while prednisolone was used to reduce inflammation and nerve oedema. Corticosteroids are the primary treatment, with a standard 60 to 80 mg daily regimen for approximately one week [4]. Evidence reveals that combined corticosteroids and antivirals can improve outcomes compared with using corticosteroids alone [4].

Bilateral facial nerve palsy has been described in association with a wide range of congenital abnormalities (Möbius syndrome, myotonic dystrophy), cranial trauma, infections (Lyme disease, Guillain-Barré syndrome, herpes zoster, syphilis, leprosy, infectious mononucleosis, influenza, mumps, chickenpox, poliomyelitis, AIDS), metabolic disorders (acute porphyria, diabetes mellitus) and neoplasms (myeloproliferative syndromes, von Recklinghausen disease) [5]. Causes other than viral diseases, such as vasospasm, vascular insufficiency and autoimmune phenomena, have also been reported. The diagnosis of Bell’s palsy should only be made once other possible causes have been excluded [5].

The pathophysiology of Bell's palsy is not entirely understood but is believed to involve viral-induced inflammation and ischemia of the facial nerve. The role of the herpes simplex virus in the development of Bell's palsy is supported by the detection of viral DNA in endoneurial fluid and saliva of affected individuals [16].

The management of bilateral facial nerve palsy involves a multidisciplinary approach, including neurologists, ENT specialists, and ophthalmologists, to address the various aspects of the disease. In cases of incomplete eye closure, ophthalmologists play a crucial role in preventing corneal damage through protective measures. ENT specialists may provide further evaluation and management of persistent or refractory cases.

Despite the rarity of bilateral Bell's palsy, this case underscores the importance of recognizing it as a potential diagnosis in patients presenting with facial weakness. Early intervention with corticosteroids can significantly improve outcomes. Referral to specialists, such as ENT and ophthalmology, is essential for comprehensive management and monitoring of potential complications.

## Conclusions

This case of idiopathic bilateral Bell's palsy in a 31-year-old male highlights the challenges and considerations in diagnosing and managing rare causes of facial paralysis. The patient's presentation was characterized by progressive bilateral facial weakness following neck and jaw pain, and the subsequent normal findings on routine tests and imaging supported the diagnosis of bilateral Bell's palsy. The prompt administration of acyclovir and prednisolone and the arrangement for specialist follow-up reflect the standard of care in such cases. This report adds to the limited literature on bilateral Bell's palsy, emphasizing the importance of considering it in the differential diagnosis and the efficacy of early treatment.

In summary, bilateral Bell's palsy, though rare, should be considered in patients with acute bilateral facial paralysis. Comprehensive evaluation to exclude other potential causes is essential, and early treatment with corticosteroids remains the cornerstone of management. This case demonstrates the importance of a multidisciplinary approach in diagnosing and managing Bell's palsy, ensuring optimal patient outcomes and preventing long-term complications.
